# Localization with Transfer Learning Based on Fine-Grained Subcarrier Information for Dynamic Indoor Environments

**DOI:** 10.3390/s21031015

**Published:** 2021-02-02

**Authors:** Yuqing Yin, Xu Yang, Peihao Li, Kaiwen Zhang, Pengpeng Chen, Qiang Niu

**Affiliations:** 1 China Mine Digitization Engineering Research Center, Ministry of Education, Xuzhou 221116, China; yinyuqing@cumt.edu.cn (Y.Y.); yang_xu@cumt.edu.cn (X.Y.); chenp@cumt.edu.cn (P.C.); 2 School of Computer Science and Technology, China University of Mining and Technology, Xuzhou 221116, China; ts18170012a31@cumt.edu.cn (P.L.); 8182879@cumt.edu.cn (K.Z.)

**Keywords:** indoor localization, channel state information, transfer learning, multi-domain representations

## Abstract

Indoor localization provides robust solutions in many applications, and Wi-Fi-based methods are considered some of the most promising means for optimizing indoor fingerprinting localization accuracy. However, Wi-Fi signals are vulnerable to environmental variations, resulting in data across different times being subjected to different distributions. To solve this problem, this paper proposes an across-time indoor localization solution based on channel state information (CSI) fingerprinting via multi-domain representations and transfer component analysis (TCA). We represent the format of CSI readings in multiple domains, extending the characterization of fine-grained information. TCA, a domain adaptation method in transfer learning, is applied to shorten the distribution distances among several CSI readings, which overcomes various CSI distribution problems at different time periods. Finally, we present a modified Bayesian model averaging approach to integrate the multi-domain outcomes and give the estimated positions. We conducted test-bed experiments in three scenarios on both personal computer (PC) and smartphone platforms in which the source and target fingerprinting data were collected across different days. The experimental results showed that our method outperforms state-of-the-art methods in localization accuracy.

## 1. Introduction

Indoor localization systems play increasingly important roles in many emerging applications, such as indoor navigation [1,2], disaster rescue [3], elderly care [4], etc. [5]. Due to the ubiquitous Wi-Fi devices in office and home environments, Wi-Fi-based device-free localization has attracted a lot of research interest [6,7]. Manikanta Kotaru et al. [8] proved that Wi-Fi localization can achieve decimeter-level positioning accuracy. The existing positioning works based on Wi-Fi can be roughly divided into two methods: signal propagation modeling [9,10,11] and radio frequency fingerprinting [12,13,14,15,16]. The former, a range-based method, studies the signal propagation path and noise distribution to establish a formula for the relationship between the signal intensity and distance of transmitter/receiver, i.e., a signal propagation loss model, and finally calculates the positioning coordinates by means of triangulation, such as with time of flight and angle of arrival. The latter method can be roughly divided into two stages: an offline training stage and an online positioning stage. The offline phase requires the manual acquisition of Wi-Fi signal values, such as the received signal strength indicator (RSSI) or channel state information (CSI), received at different locations to construct a fingerprint database. In the online positioning stage, an unknown position is predicted by matching the received signal value and the signal value in the fingerprint database.

However, a problem has been neglected: In a dynamic indoor environment, the offline information in one time period in the fingerprint database and the online information observed in a later time period cannot follow the same distribution due to the fact that Wi-Fi is vulnerable to environmental dynamics due to path attenuation, occlusion, and multipath effects. Therefore, it is particularly important to design an indoor positioning method to adapt to the dynamic environment. A feasible strategy is to readjust the radio map, but this requires huge human and financial resources. Another way to cope with the change in signal distribution in the time domain is transfer learning [17,18], which adapts the localization model trained in one time period (the source domain) to a new time period (the target domain). Transfer learning can overcome the influence of the dynamic environment on signal change. Therefore, this paper aims to propose an indoor localization method that integrates multi-domain representation and transfer learning.

To study the various possibilities of Wi-Fi signals, this paper collects CSI signals to construct a fingerprint dataset. Compared with the RSSI, CSI can provide higher-dimensional and more fine-grained information [19,20]. In addition, compared with other papers using multiple APs (access points) to construct an RSSI reading matrix, this paper collects only CSI signals between a single AP and mobile devices to build the fingerprint dataset. To compensate for the relative lack of information caused by using a single AP, this paper proposes three domain representation methods for CSI signals, namely, the amplitude of the time domain, the transform in the wavelet domain, and the relative distance in the graph domain, which enrich the numerical representation of CSI signals from different perspectives. To further eliminate the influence of dynamic environmental changes on Wi-Fi fingerprint signals and reduce the workload of re-calibrating fingerprint databases, an indoor location method based on multi-domain representation and transfer learning is proposed in this paper. First, we remove the outliers from and smooth the CSI fingerprint data collected in two different time intervals (source domain and target domain). Then, we transform them into multi-domain representations. We use the idea of transfer learning to map the data from the corresponding source and target domains to the latent feature space, and use the KNN (k-nearest neighbor) algorithm to align and match the unlabeled target-domain data and the labeled source-domain data. For the predicted labels in the three fields, the weighted centroid algorithm is used to calculate the predicted coordinates of the target points. We collected CSI data from different scenarios over two different periods on two days. A real experiment was used to verify the validity of the transfer learning idea in CSI fingerprint classification, and the highest label prediction accuracy in the target domain could reach 1. In addition, we compare the localization method, the traditional support vector machine (SVM) algorithm, and the deep learning algorithm. The results show that our algorithm can reach a decimeter-level average localization error.

To sum up, the main contributions of this paper are as follows:

1. To overcome the variations in Wi-Fi signals across time, we apply transfer learning to CSI readings for indoor fingerprinting localization.
2. Unlike using one feature as a fingerprint, we propose three types of CSI feature representations-amplitude in the time domain, wavelet transformations in the frequency domain, and similarity distance in the shape domain-which make full use of the CSI characters in three domains. We also propose a novel strategy utilizing Bayesian model averaging and the weighted centroid algorithm to better fuse the localization results corresponding to the three features.
3. We collected CSI data from a computer equipped with an Intel WiFi Link 5300 wireless network interface card and conducted two experiments in an open hall and a laboratory. The proposed method’s effectiveness and robustness were verified by comparing the traditional CSI fingerprinting location method with different algorithm parameters.
4. We also evaluated our method for the efficiency and validity of localization across several days on a smartphone platform. The experimental results show that the proposed system can achieve performance on a smartphone platform that is comparable with that on the computer platform and is characterized by good robustness.

The remaining chapters of this paper are arranged as follows: Section 2 describes our motivation with experimental data. Section 3 introduces the overall design of the location algorithm. Section 4 reports the experimental results of localization algorithms in two scenarios. The last section summarizes the whole paper.

## 2. Motivation

This section illustrates the motivation of this algorithm with the help of real experimental data. First, we analyze the reasons for choosing CSI instead of the RSSI as the Wi-Fi information to be read. Then, the dynamic performance of CSI over time is used to show the necessity of transfer learning theory. Finally, we provide pre-observation remarks by comparing the waveform to the algorithm design.

### 2.1. RSSI vs. CSI

In the last two decades, a large number of applications using signal intensity information to perceive environmental characteristics have emerged, among which the most representative one is wireless indoor positioning based on the RSSI value [21,22,23]. Note that RSSI is processed and scaled by the actual received signal strength and it is always a positive value to make it easy to understand. However, in a single-AP scenario, an RSSI-based indoor location algorithm contains the following two limitations: (1) Triangulation requires at least three APs to locate coordinates, and (2) if an RSSI fingerprint location is used, its characteristic dimension is only 1. Environmental changes and multi-path propagation can easily cause RSSI fluctuations and lead to incorrect fingerprint matching of wireless signals.

Researchers have modified firmware to obtain a sample version of the CFR (channel frequency response, including the amplitude-frequency response and the phase-frequency response) in the form of CSI on ordinary Wi-Fi devices. Specifically, a set of CSI can be obtained from each received packet by using a wireless network card compatible with IEEE 802.11a/g/n. Each CSI group represents the magnitude and phase of an orthogonal frequency division multiplexing (OFDM) subcarrier [24].

Compared with the RSSI, CSI expands the amount of wireless channel information. In a single-AP environment, it can provide multidimensional feature information fingerprints, which improve the recognition of signals received at different locations by the receiver and are more conducive to indoor fingerprint matching and positioning [25,26]. In addition, existing works have studied the application of the transfer learning method to RSSI reading. This paper studies the application of a transfer learning method to indoor positioning using CSI.

### 2.2. Limitations and Opportunities

**Figure 1:**
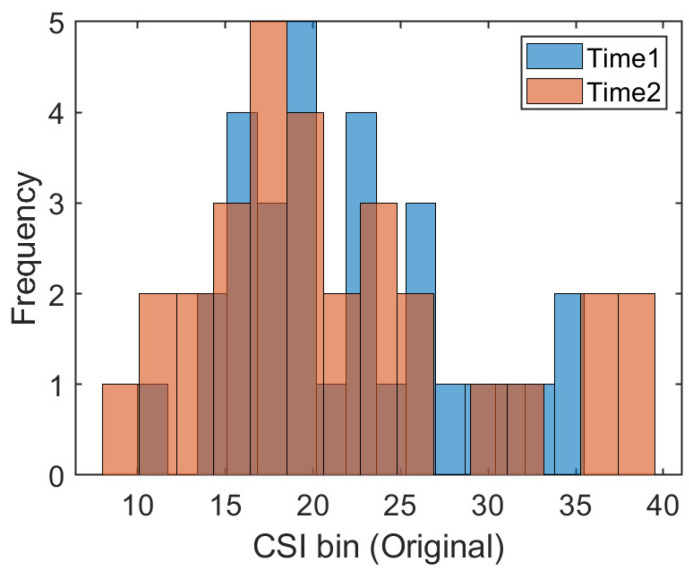
The frequency histograms at position #1 during the two time periods.

The traditional fingerprint-based machine learning localization algorithm assumes that the training data and test data follow the same distribution. The well-trained CSI mapping model is directly applied to position estimation in different time periods. In addition, some works, such as that of [27], have collected data on different dates as the training set to eliminate the influence of CSI changes across time. However, in a changing indoor environment, the occlusion of obstacles, the interference of radio electromagnetic signals, and the unpredictable motion of moving objects make the CSI uncertain. So, it can not satisfy the assumption of the same distribution in most cases. To provide visual evidence, we collected several CSI readings at the same position in two different time periods. We drew a frequency histogram to illustrate the distribution of the amplitude data of the 30 subcarriers, which is shown in Figure 1. The X-axis represents the bins of CSI amplitudes and the Y-axis represents the statistical frequency of the amplitudes within each bin. It is obvious that CSI readings are subject to different distributions at the same position under different times. One promising solution is to collect training data in different periods to eliminate some dynamic effects while increasing the amount of manual labor in the acquisition process. For the problems related to the different distributions of CSI in different time periods and the large amount of manual labor required to acquire data, this paper applies transfer learning to realize adaptive fingerprinting localization using the potential spatial alignment of the data collected in the previous time period and the data collected in the later time period.

**Figure 2:**
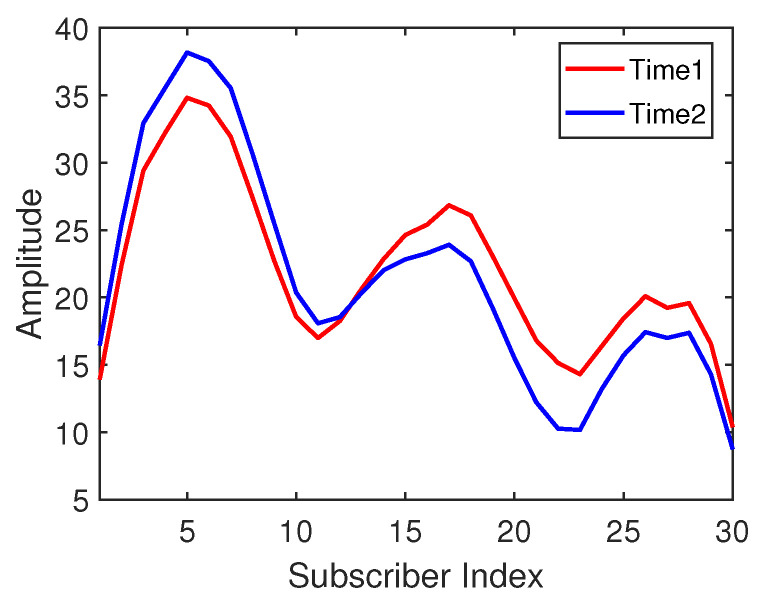
The amplitude waveforms at position #1 under two time periods.

It is discussed that the amplitude distribution of CSI subcarriers is different in different time periods. However, from another point of view, their waveforms may have some similarity. Figure 2 shows the CSI waveform acquired by an antenna at the same location (the same in Figure 1) in two different time periods. The x-axis represents the index of 30 subcarriers, and the y-axis represents the amplitude of the subcarriers. It can be seen that the change trends of the subcarrier waveforms collected at the same location point at time 1 and time 2 are similar. Therefore, this intuitive phenomenon inspires us to propose a waveform representation method to calculate the similarity between the two waveforms. This new waveform similarity representation will be used as a kind of CSI fingerprint identification method to process and classify the information in the transfer learning method.

## 3. Methodology

**Figure 3:**
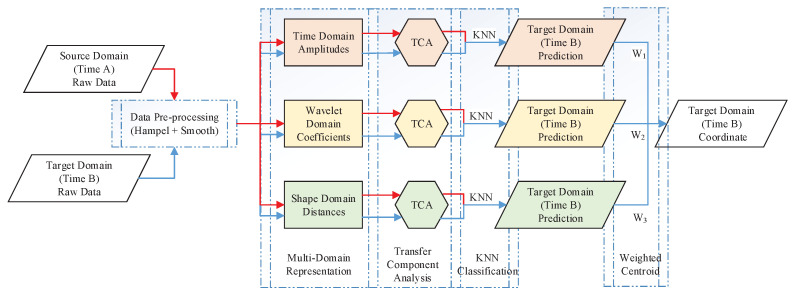
Systematic method diagram.

The system’s methodological structure is shown in Figure 3. First, the CSI source data collected at different locations in different time periods are preprocessed. Second, multi-domain representation processing is carried out separately for each domain. The data of the source domain and target domain are mapped into the potential feature space through transfer component analysis (TCA), and the predictive location label of the target-domain data is labeled with the KNN algorithm. Finally, the predicted coordinates of the target point are obtained through the weighted centroid positioning of the three position prediction results. Therefore, we name the proposed approach LMDR-TCA (localization with multi-domain representation and TCA). Next, each module of the system will be described in detail in this section.

### 3.1. Data Statement

A Wi-Fi router is deployed as a transmitter in the indoor environment. At a certain moment *a*, an original CSI signal received by mobile terminals, such as an antenna, a computer, or a mobile phone, at position *i* is recorded as $CSI_{a}^{i}=\left(s_{real}^{1},s_{imag}^{1},...,s_{real}^{N},s_{imag}^{N}\right)$. Each original CSI signal datum contains *N* sets of subcarrier complex pairs. The real part is $s_{real}^{N}$ and the imaginary part is $s_{imag}^{N}$. The original signal collected in period *A* is recorded as matrix ${\mathrm{CSI}}_{A}^{i}={\left(CSI_{a1}^{i},CSI_{a2}^{i},\ldots ,CSI_{am}^{i}\right)}^{T}$. *m* is the number of data packets per fingerprint point, which is calculated by the product of the sampling time *t* and sampling frequency *f*. In the original environment, CSI signals at different locations are collected as fingerprint databases, which are also called the source-domain dataset, and are recorded as $D_{S}={\left\{\left({\mathrm{CSI}}_{S}^{i},l_{i}\right)\right\}}_{i=1}^{I}$, where $l_{i}$ is the label of position *i* and *I* is the number of fingerprint points collected in the original environment. When the indoor environment changes or dynamic changes occur, the data of the points to be measured without location information are collected; they are recorded as the dataset of the target domain and are named $D_{T}={\left\{\left({\mathrm{CSI}}_{T}^{i}\right)\right\}}_{j=1}^{I}$. Then, our goal is to align the data of the source domain and the target domain in the potential feature space using transfer learning and to label the target domain with a location tag.

### 3.2. Data Preprocessing

Due to the inherent noise that influences the receiver, we need to preprocess the fingerprint signals collected over a period of time, which includes outlier removal and smoothing. For the signal matrix ${\mathrm{CSI}}_{A}^{i}$ that is acquired in time period *A* at position point *i*, the Hampel identifier is used to remove the outliers. For the n-th subcarrier complex, a point outside of the closed interval $\left[\mu_{n}-\gamma *\sigma_{n},\mu_{n}+\gamma *\sigma_{n}\right]$ is defined as an outlier, where $\mu_{n}$ is the median of the sequence, $\sigma_{n}$ is the median absolute deviation (MAD) of the sequence, and γ is generally set at the default value of 3. Then, we add the median value at the outlier point. The reason for why we choose median and MAD instead of the commonly used mean and standard deviation is because the latter two parameters are extremely sensitive to the presence of outliers in the data. Then, a low-pass filter using the moving average method is applied to smooth the sequence, where the sliding window size is 5 and the filtering coefficient is reciprocal of the sliding window size.

### 3.3. Multi-Domain Representation

Preprocessed CSI data can be processed from various different perspectives for multi-domain representations. This work especially considers two commonly used aspects, CSI amplitudes and wavelet transformations, as well as a novel aspect using the shape correlation.

#### 3.3.1. Time-Domain Amplitudes

**Figure 4:**
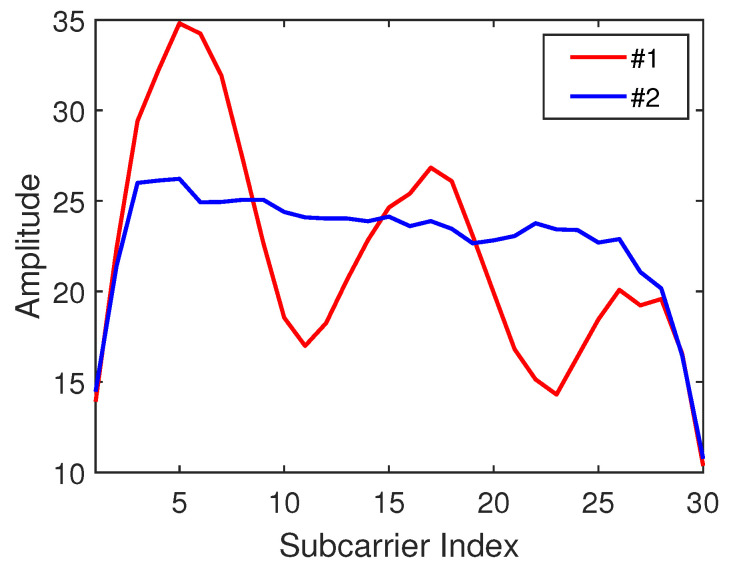
Channel state information (CSI) amplitude waveform of adjacent nodes.

The subcarrier amplitude sequences at different positions in the time domain can be considered as the most classical fingerprint features. Unlike similar values of adjacent points of an RSSI signal, the CSI signals of adjacent points may be quite different. Figure 4 shows the CSI subcarrier amplitude waveforms received at two different locations 1.2 m apart in the same time period. Therefore, the CSI amplitude as a feature of the location fingerprint has a greater degree of identification for different location information. To be more specific, the formula for calculating the amplitude of a CSI signal is as follows:

(1) $$\begin{array}{cc} CSI_{a_{-}amp}^{i} & =\left\{S_{a}^{i1},S_{a}^{i2},\ldots ,S_{a}^{iN}\right\}\\ & =\{\sqrt{{\left(s_{real}^{1}\right)}^{2}+{\left(s_{imag}^{1}\right)}^{2}},\ldots ,\sqrt{{\left(s_{real}^{N}\right)}^{2}+{\left(s_{imag}^{N}\right)}^{2}}\}. \end{array}$$

In summary, the source and target domains after amplitude calculation are as follows:

$D_{s_{-}amp}={\left\{\left({\mathrm{CSI}}_{S_{-}\mathrm{amp}}^{i},l_{i}\right)\right\}}_{i=1}^{l}$
$D_{T_{-}amp}={\left\{\left({\mathrm{CSI}}_{T_{-}\mathrm{amp}}^{i}\right)\right\}}_{i=1}^{I}$

Among them, ${\mathrm{CSI}}_{S_{-}\mathrm{amp}}^{i}$ and ${\mathrm{CSI}}_{T_{-}\mathrm{amp}}^{i_{\underline{r}}}$ are composed of a characteristic matrix in the form of (I×m) rows × *N* columns, where *I* is the number of fingerprint points, *m* is the number of data packets sampled for each fingerprint point, and *N* is the subcarrier dimension.

#### 3.3.2. Wavelet-Domain Transformations

However, another powerful information extraction tool is the wavelet transformation, which is able to capture the local fluctuations and abrupt changes of a signal. The object of the wavelet transform is the amplitude data of each subcarrier in the time domain. We use the “db4” wavelet to decompose three layers and apply soft threshold processing for each layer detail coefficient (high frequency coefficient). Finally, we reconstruct the wavelet system layer by layer to get the wavelet transform signal. In summary, the source domain and target domain after the wavelet transform are, respectively, $D_{s_{-}wave}={\left\{\left({\mathrm{CSI}}_{S_{-}{wave}^{\prime }}^{i},l_{i}\right)\right\}}_{i=1}^{I}$ and $D_{T_{-}wave}={\left\{\left({\mathrm{CSI}}_{T_{-}wave}^{i}\right)\right\}}_{i=1}^{I}$. Here, ${\mathrm{CSI}}_{S_{-}wave}^{i}$ are composed of the (I×m)×N characteristic matrix, *I* is the number of fingerprint points, *m* is the number of data packets sampled for each fingerprint point, and *N* is the subcarrier dimension.

#### 3.3.3. Shape-Domain Distances

Based on the findings of the previous section, it is believed that the similarity between CSI amplitude waveforms at two locations can be used as CSI fingerprint information at those locatiosn to match the same location information at different times. Generally speaking, the similarity distance between two CSI amplitude waveforms can be calculated by the dataset $P=\left({\mathrm{CSI}}_{a_{-}\mathrm{amp}}^{i},{\mathrm{CSI}}_{b_{-}\mathrm{amp}}^{r},S_{a_{-}pair}^{i},S_{b_{-}pair}^{r}\right)$. $S_{a_{-}pair}^{i},S_{b_{-}pair}^{r}$ are the sets of subcarrier sequence pairs for each CSI sequence. $S_{a_{-}pair}^{i}=\left\{\left(S_{a}^{i1},S_{a}^{i2}\right),\left(S_{a}^{i1},S_{a}^{i3}\right),\ldots ,\left(S_{a}^{iN-1},S_{a}^{iN}\right)\right\}$, $S_{b_{-}pair}^{r}=\left\{\left(S_{b}^{R_{1}},S_{b}^{R_{2}}\right),\left(S_{b}^{R_{1}},S_{b}^{R_{3}}\right),\ldots ,\left(S_{b}^{rN-1},S_{b}^{rN}\right)\right\}$, and the number of sequence pairs in a set is $C_{N}^{2}$. Next, for the sequence pairs corresponding to two sets $\left(S_{a}^{ix},S_{a}^{iy}\right)\in S_{a_{-}pair}^{i}$ and $\left(S_{b}^{\prime x},S_{b}^{\gamma y}\right)\in S_{b_{-}pair}^{r}$, if $S_{a}^{k}<S_{a}^{f}$ and $S_{b}^{rx}<S_{b}^{\eta y}$ (or $S_{a}^{ix}>S_{a}^{iy}$ and $S_{b}^{rx}<S_{b}^{\eta }$ ), we refer to these corresponding sequence pairs as sequential sequence pairs, the number of which is denoted as $Q_{rev}$. If $S_{a}^{ix}<S_{a}^{iy}$ and $S_{b}^{rx}>S_{b}^{ry}$ (or $S_{a}^{i\kappa }>S_{a}^{iy}$ and $S_{b}^{rx}<S_{b}^{ry}$), then we call this an inversion sequence pair, and the number of inversion sequence pairs is denoted as $Q_{rev}$. Then, $C_{N}^{2}=Q_{ovd}+Q_{rw}$. The similarity distance between two CSI amplitude waveforms is calculated by dividing the number of inversion sequence pairs by the number of sequence pairs of the whole set:

(2) $$D\left({\mathrm{CSI}}_{a_{-}\mathrm{amp}}^{i},{\mathrm{CSI}}_{b_{-}\mathrm{amp}}^{r}\right)=\frac{Q_{rev}}{C_{N}^{2}}.$$

In summary, the distances calculated by pairs constitute the feature matrix of (I×m)×I.

### 3.4. Transfer Component Analysis

Transfer component analysis (TCA) [28] aims to find a suitable mapping ϕ that minimizes the distance between the source-domain data distribution and the target-domain data distribution after mapping. Let $n_{1}$ and $n_{2}$ denote the number of samples in the source domain and target domain, respectively. Taking the time-domain amplitude as an example, we use maximum mean discrepancy (MMD) [29] distance to define the distance between them.

(3) $$\begin{array}{c} DISTANCE(D_{S\_amp},D_{T\_amp})\\ ={∥\frac{1}{n_{1}}\sum_{i=1}^{n_{1}}\phi (CSI_{S\_amp\_i})-\frac{1}{n_{2}}\sum_{i=1}^{n_{1}}\phi (CSI_{T\_amp\_i})∥}_{H} \end{array}$$

Since we can not directly find the appropriate mapping, we introduce a kernel function *K* and an MMD matrix *L* to transform the difficult distance into a solvable one, that is,

(4) $$\mathrm{DISTANCE}\left(D_{S_{-arm}p}D_{T_{-amp}}\right)=\mathrm{tr}\left(KL\right)-\lambda ,\mathrm{tr}\left(K\right)$$

where

(5) $$K=\left[\begin{array}{cc} K_{S,S} & K_{S,T}\\ K_{T,S} & K_{T,T} \end{array}\right]$$

(6) $$L_{ij}=\left\{\begin{array}{cc} \frac{1}{n_{1}^{2}} & CSI_{S_{-}amp_{-}i},CSI_{S_{-}arp_{-}j}\in D_{S_{-}arp}\\ \frac{1}{n_{2}^{2}} & CSI_{I_{-}amp_{-}i},CSI_{T_{-}arp_{-}j}\in D_{T_{-}axp}\\ -\frac{1}{n_{1}n_{2}} & \mathrm{otherwise}\; \end{array}\right.$$

Here, tr() represents the trace of a matrix, that is, the sum of the diagonal elements of a matrix.

Since this is a semi-definite programming (SDP) problem in mathematics, the dimensionality reduction method is used to optimize the target distance equation. We use the m-dimensional matrix *W* to construct the transformation form of the kernel function:

(7) $$\tilde{K}=\left(KK^{-1/2}\tilde{W}\right)\left(W^{T}K^{-1/2}K\right)=KWW^{T}K$$

Here, $W=K^{-1/2}\tilde{W}$. Substituting it into Equation (4), the ultimate optimization goal of the distance between the source domain and the target domain can be obtained:

(8) $$\begin{array}{c} \min_{W}\mathrm{tr}\left(W^{T}KLKW\right)+\mu \mathrm{tr}\left(W^{T}W\right)\\ \;s.t.\;W^{T}KHKW=I_{m} \end{array}$$

where μ is the trade-off parameter, $I_{m}$ is an identity matrix of m×m, $H=I_{n_{1}+n_{2}}-1/\left(n_{1}+n_{2}\right)11^{T}$ is a central matrix, and 1 is a column vector with a length of $n_{1}+n_{2}$ where the elements are all 1. The above formula contains a non-convex norm constraint that can be transformed into Lagrangian dual form:

$$\;\;\;\;\;\;\;\;\;\;\;\;\;\;\;\;\;\;\;\;\;\;\;\;\;\;\;\;\min_{W}\mathrm{tr}\left({\left(W^{T}KHKW\right)}^{†}W^{T}\left(I+\mu KLK\right)W\right)$$

or an equivalent trace maximization problem:

$$\;\;\;\;\;\;\;\;\;\;\;\;\;\;\;\;\;\;\;\;\;\;\;\;\;\;\;\;\max_{W}\mathrm{tr}\left({\left(W^{T}\left(I+\mu KLK\right)W\right)}^{-1}W^{T}KHKW\right).$$

The solution of *W* in the formula is comprised of the eigenvectors corresponding to the *m* leading eigenvalues of ${\left(I+\mu KLK\right)W)}^{-1}W^{T}KHK$.

**Figure 5:**
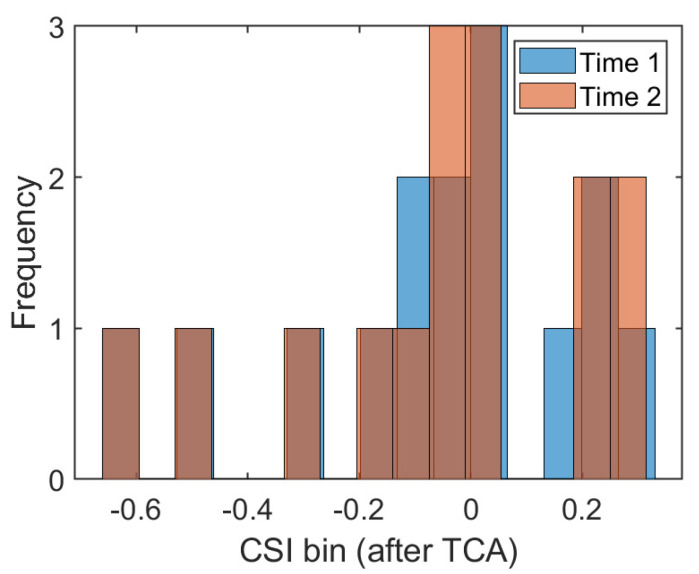
Frequency histogram after transfer component analysis (TCA) transformation at position #1 (m = 15).

From this, the dimensionality reduction data of the source domain and target domain are expressed as $D_{S_{-amp}}^{\prime }$ and $D_{T_{-amp}}^{\prime }$. Figure 5 shows the frequency histogram of the position #1 amplitude in two different time periods transformed via TCA and reduced by dimension. Compared with Figure 1, the data distribution after dimensionality reduction through TCA transformation is more similar, which also proves that the idea of transfer learning has some advantages in closing the data distribution distance of different domains.

### 3.5. Label Alignment

After the TCA transformation, the characteristic matrices of the source domain and target domain are respectively obtained. We use the traditional KNN algorithm to align the characteristics of the source domain and target domain, that is, to identify the category of the target-domain data, and finally affix the label of the aligned source-domain data to the corresponding prediction data of the target domain. The specific ideas of the KNN are as follows: If most of the *k* similar samples in the feature space (i.e., the nearest neighbors in the feature space) belong to a certain category, the sample also belongs to this category. Similar distances here are measured using the city block distance, which is the sum of the absolute differences between two eigenvectors, and can be expressed as follows. *s* and *t* are each an eigenvectors of the source domain or the target domain, respectively, and *m* is the dimension after TCA transformation of the eigenvector.

(9) $$d\left(s,t\right)=\left|s_{1}-t_{1}\right|+\left|s_{2}-t_{2}\right|+\ldots +\left|s_{m}-t_{m}\right|$$

### 3.6. Localization Estimation

The data in the target domain contain an amount of information for a period of time (1 min) for each location to be measured. According to the sampling rate, we can conclude that the amount of information for each location point to be measured is on the order of thousands of eigenvectors. According to the KNN method, thousands of location labels of the location to be measured can be obtained. In addition, we obtain the labels to be measured through three expressions. In this section, we will introduce the weight calculation of each expression and the coordinate calculation method of each position to be measured.

For one target-domain signal, the representation model $M^{k}\left(K=1,2,3\right)$ generates a prediction label $P\left(l_{tar}^{i}|M^{k},CSI_{tar}\right)$ for it. We use the Bayesian model averaging approach [30] to fuse the predicted results of three representations and get the probability formula that the target signal predicts for position i:

(10) $$P\left(l_{tar}^{i}|CSI_{tar}\right)=\sum_{k=1}^{K}P\left(l_{tar}^{i}|M^{k},CSI_{tar}\right)\times P\left(M^{k}|CSI_{tar}\right).$$

Here, $P\left(M^{k}|CSI_{tar}\right)$ is defined as the weight of the *k*-representation model, and its representation method is as follows:

(11) $$P\left(M^{k}|CSI_{tar}\right)=w^{k}=\frac{1}{2}\times \frac{\sum_{j=1,j\neq k}^{K}\mathrm{Corr}\left(M^{k},M^{j}\right)}{\sum_{i=1}^{K}\sum_{j=1,j\neq i}^{K}\mathrm{Corr}\left(M^{i},M^{j}\right)}.$$

$\mathrm{Corr}\left(M^{i},M^{j}\right)$ represents the correlation coefficients of the result matrix using *i* to represent the model prediction and *j* to represent the result matrix of the model prediction, which can be calculated by the square root of the covariance coefficient of the two divided by the product of their variance:

(12) $$\mathrm{Corr}\left(M^{i},M^{j}\right)=\frac{\mathrm{Cov}\left(M^{i},M^{j}\right)}{\sqrt{\mathrm{Var}\left(M^{i}\right)\times \mathrm{Var}\left(M^{j}\right)}}.$$

Finally, the predicted position of the node can be calculated with the weighted centroid algorithm:

(13) $$pos=\sum_{i=1}^{L}P\left(l_{tar}^{i}|CSI_{tar}\right)\times pos_{true}^{i},$$

where $pos_{true}^{i}$ represents the real coordinates of the *i*-position nodes, $P\left(l_{\mathrm{tor}}^{i}|CSI_{\mathrm{tar}}\right)$ represents the prediction probability of position *i*, and *L* is the number of fingerprint positions in the scene.

## 4. Experiments

This section mainly introduces the experimental results of positioning in two indoor scenarios, namely, an open hall and a laboratory environment full of computers. In each experimental environment, we selected two different time intervals ($T_{1}$ and $T_{2}$) for two consecutive days as the original data of the source and target domains, where $T_{1}$ is earlier than $T_{2}$. During the experiment, we show both the transfer location results from $T_{1}$ to $T_{2}$ and the transfer location results from $T_{2}$ to $T_{1}$. The conversion experiment of the source domain and target domain can better reflect the randomness of the CSI in time, as well as the feasibility and validity of the transfer learning method in the application of CSI fingerprint location.

### 4.1. Experiment 1-Open Hall

#### 4.1.1. Data Setup

**Figure 6:**
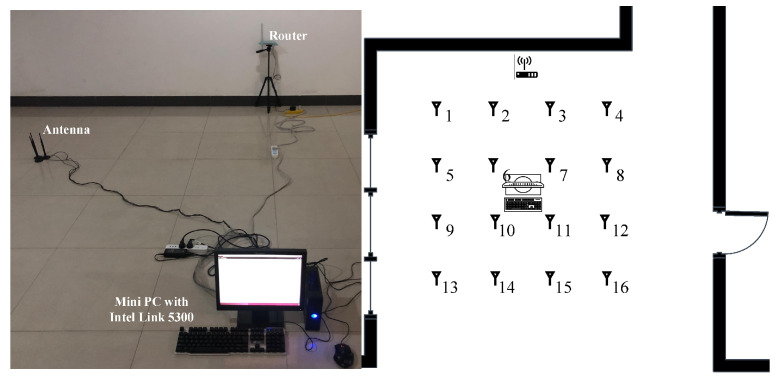
Experiment scene and floor plan of the hall.

Experiment 1 was carried out in a 7 × 10 m open hall. The transmitter end was a TP-LINK router, and the receiver end was a miniPC connecting with three omnidirectional Wi-Fi antennas at a gain of 6 dBi. The miniPC was configured with an Intel Wi-Fi Link 5300 NIC [31], which was able to control the antennas to receive wireless signals from the router and then stored raw CSI values in the firmware. The actual experimental scenario and layout are shown in Figure 6, where we moved the antennas to collect CSI values at different locations. We collected the data of 16 fingerprint points in two different periods on two consecutive days, and the distance between each fingerprint point was 1.2 m.

To measure the classification performance under different representations and the localization performance of LMDR-TCA, we define several indicators, such as the classification accuracy, localization deviation, and cumulative distribution function (CDF) of the localization deviations. For each fingerprint during $T_{1}$ or $T_{2}$, about 4000 packets of CSI signals were gathered to form the source or target datasets. The classification accuracy is the ratio of the number of correctly labeled packets to the total number of packets in the target dataset. The localization deviation can be calculated as the Euclidean distance between the actual location coordinates and the estimated location coordinates, and its units are meters. The CDF represents the accumulated probabilities under different localization deviations.

#### 4.1.2. Classification Results of Transfer Learning under Different Representations

**Figure 7:**
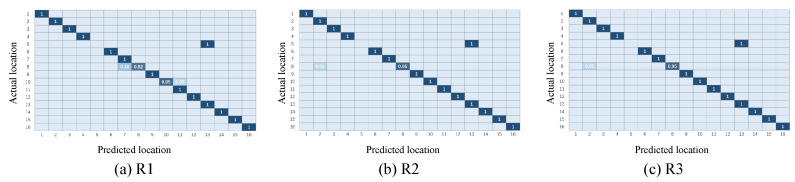
Confusion matrix under different multi-domain representations ($T_{1}$-$T_{2}$).

**Figure 8:**
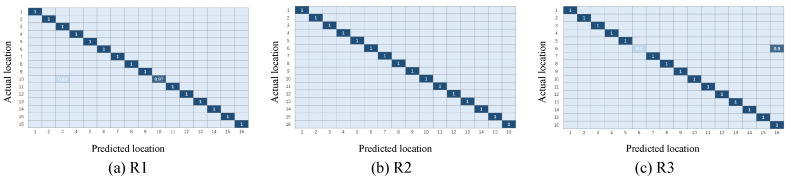
Confusion matrix under different multi-domain representations ($T_{2}$-$T_{1}$).

This section will show the classification results of the transfer learning method under the three representations and will initially confirm the effectiveness of the three representations, as well as the necessity of the combination of the three representations. Figure 7 and Figure 8 illustrate the confusion matrix of the classification results under different multi-domain representations. It is noted that in the rest of this paper, we use $R_{1}$ to represent the time-domain amplitude, $R_{2}$ to represent the wavelet-domain transformation, and $R_{3}$ to represent the shape-domain distance. In addition, $T_{1}$-$T_{2}$ represents that we train the $T_{1}$ dataset as the source domain and predict the labels of the $T_{2}$ dataset. Similarly, $T_{2}$-$T_{1}$ expresses the opposite meaning. In these two groups of figures, the location indexes of the ground truth and prediction are arranged on the *y*-axis and *x*-axis, respectively. We color each block to represent the classification accuracy of each position point and fill the specific values of accuracy in the blocks. In addition, the blank slots indicate an accuracy of 0. It can be inferred from Figure 7 and Figure 8 that: (i) among the three different representations, almost all points are sorted into the correct positions, since the diagonals are almost blue blocks; (ii) a few blocks out of the diagonals illustrate the complementary phenomenon in which the point is sorted to the incorrect category under one or two representations, while this point is correctly classified under the remaining representations. Taking Figure 7 as an example, the point #5 is completely misclassified as point #13 under $R_{1}$ and $R_{2}$, while $R_{3}$ provides the exact classification result. Instead, the point #13 is confused with the point #5 under $R_{3}$, but it is identified in the correct position under $R_{1}$ and $R_{2}$. The complementarity may make it possible to improve the final localization accuracy to some extent through our proposed weighted centroid method. The follow-up experiments will further verify the effectiveness of the combination of the three representations at improving the localization performance.

#### 4.1.3. Comparison with Different Methods

This section will present the results of the comparison between the transfer learning method and other methods with respect to the classification accuracy and localization performances, aiming at showing the wise choice of the TCA method. The comparison methods are KNN, SVM (support vector machine), and GFK (geodesic flow kernel) [32]. It should be noted that the KNN approach removes the process of TCA in the proposed system and directly calculates the nearest neighbor as the prediction. SVM, the traditional machine learning method, uses a kernel function transform like in the same step in TCA; thus, we unify the kernel function as a polynomial kernel. GFK proposes a new kernel-based method that takes advantage of low-dimensional structures, and it models domain shift by integrating an infinite number of subspaces that characterize changes in geometric and statistical properties from the source to the target domain.

**Figure 9:**
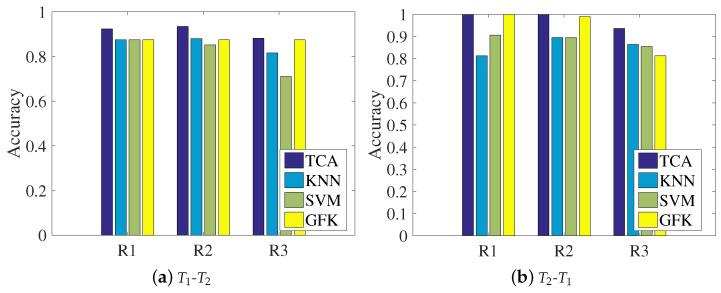
Chart of the comparison of classification accuracy among different representations: (**a**) training with the $T_{1}$ dataset and testing on the $T_{2}$ dataset; (**b**) training with the $T_{2}$ dataset and testing on the $T_{1}$ dataset.

Figure 9 shows the localization accuracies under the three representations for the four methods, where the left subfigure regards the $T_{1}$ dataset as the source domain and the $T_{2}$ dataset as the target domain, and the right subfigure shows the opposite condition. It can be clearly seen that for each representation, the data transferred by our chosen TCA method can be aligned better than with the other methods, since the classification accuracy of TCA is the highest among the comparison approaches. In addition, there are performance differences between the two transfer learning methods of TCA and the GFK. In TCA, the source domain and target domain are simultaneously mapped into the reproducing kernel Hilbert space, and the MMD is calculated to close the distributions of the two domains. The GFK assumes that the source domain and target domain are two points in the Grassmann manifold space, and it tries to find a geodesic line that could map the source domain to the target domain. The final results indicate that closing the distribution distance works better than finding the geodesic line, since TCA is slightly more accurate than the GFK according to the statistics.

**Figure 10:**
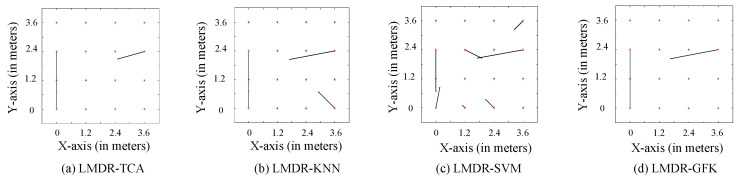
Localization performance of the different methods ($T_{1}$-$T_{2}$).

**Figure 11:**
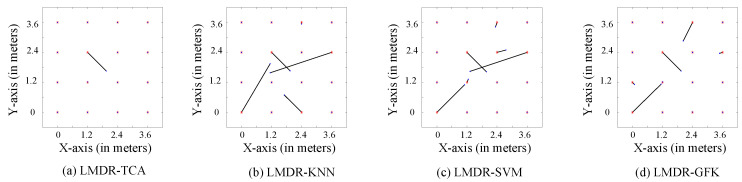
Localization performance of the different methods ($T_{2}$-$T_{1}$).

Figure 10 and Figure 11 illustrate the comparison charts of the localization performance among the four different approaches, where the final estimated coordinates are obtained by our presented fusion localization method that utilizes a modified weighted centroid algorithm to mix the classification results of the three representations. To be more specific, the red asterisks represent the ground truth points to be located in the target dataset, which are $T_{2}$ in Figure 10 and $T_{1}$ in Figure 11, blue dots show the estimated points, and black segments pointing from red asterisks to blue dots mean the localization deviations. Table 1 lists the localization performance with respect to the mean and max deviations. Compared to the LMDR-KNN, LMDR-SVM, and LMDR-GFK, LMDR-TCA achieves the best localization performance because it improves the localization accuracy by an average of 48%, 78%, and 22%, respectively. It is also noted that although the classification accuracies of TCA are almost 6–10% higher than those of the KNN, the localization performance graphs show obvious deviation differences between LMDR-TCA and LMDR-KNN. The localization error of LMDR-TCA is the smallest among the four methods, since the deviation segments shown in the subfigures are the shortest. In the open hall environment, the two transfer learning methods (LMDR-TCA and LMDR-GFK) experience deviations only to adjacent nodes; that is, an incorrectly positioned point is mostly positioned in the same row, same column, or nearest diagonal position.

**Figure 12:**
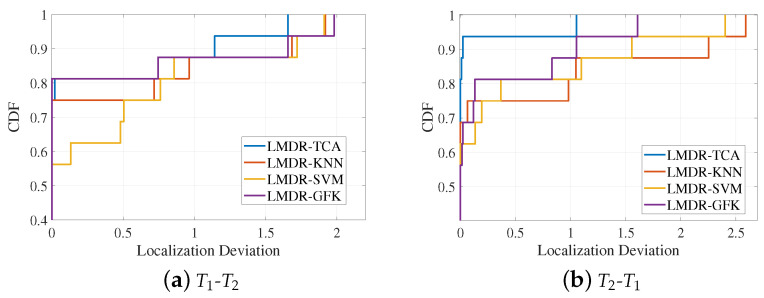
Cumulative distribution functions of localization errors: (**a**) training with the $T_{1}$ dataset and testing on the $T_{2}$ dataset; (**b**) training with the $T_{2}$ dataset and testing on the $T_{1}$ dataset.

Using the deviation values of the 16 positioning points, Figure 12 draws the curves of the cumulative distribution function (CDF) under two source-target conditions. The CDF curve can indicate the positioning accuracy under different deviation ranges. When the CDF reaches “1”, the corresponding localization deviation is the maximum deviation that can be found, as detailed in Table 1. When the localization deviation is fixed to a certain value, it can represent the percentage of points with localization errors that are less than that value. For instance, as we can see in the right panel of Figure 12, when the localization deviation is fixed to 0.25 m, the corresponding CDF values for LMDR-KNN, KMDR-SVM, LMDR-GFK, and LMDR-TCA are 0.75, 0.75, 0.8125, and 0.9375, respectively. In other words, due to the total number of positioning points being 16, the numbers of points localized at less than 0.25 m are 12, 12, 13, and 15, respectively, for the above positioning methods. Generally, the localization performance of LMDR-TCA is superior to that of the other three comparison approaches.

#### 4.1.4. Localization Performance under Different Influence Factors

This section explores the impacts of different influence factors on the localization performance for the proposed method and other comparison methods.

- Impact of the weight coefficient

**Figure 13:**
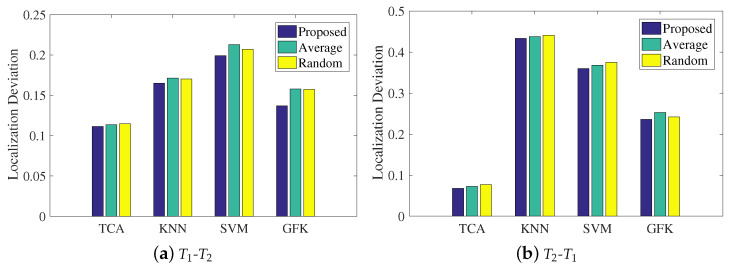
Impacts of the weighted coefficient on localization performance: (**a**) training with the $T_{1}$ dataset and testing on the $T_{2}$ dataset; (**b**) training on the $T_{2}$ dataset and testing on the $T_{1}$ dataset.

In the last step in LMDR-TCA, an improved weighted centroid method is presented to combine the three classification results related to the three types of representations in the final estimated coordinate. This subsection will explore the impact of the weight coefficient on the mean localization deviations. It is noted that the proposed method applies Equation (12) to calculate the weight coefficient, the average method uses a weight coefficient of 1/3 for each representation, and the random method randomly sets the three weight coefficients. Figure 13 illustrates the experimental results of the mean localization deviations using the different methods of calculating weight coefficients. Under both the $T_{1}$-$T_{2}$ and $T_{2}$-$T_{1}$ conditions, LMDR-TCA always results in the lowest localization deviations among the approaches. In addition, among the different methods of calculating the weight coefficients, the proposed method is a little better than the average method and the random method, which proves the effectiveness of our method. More specifically, with respect to the statistics, the proposed method improves the localization deviation by 5–10% over the other two methods.

- Impact of the combination of multi-domain representations

**Figure 14:**
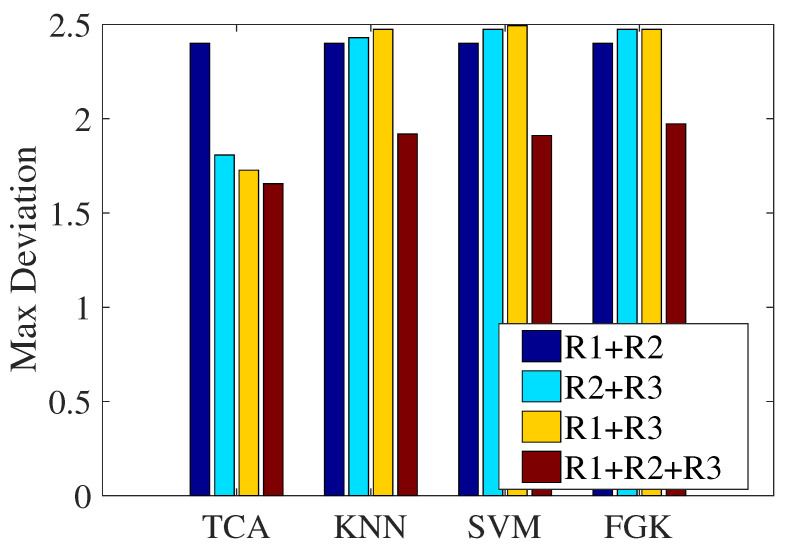
Impact of the combination of multi-domain representations on localization performance.

In the previous section, we explored the necessity of multi-domain representations by showing the confusion matrixes of the classification results of the three representations, where we can see that the three kinds of representations have complementary advantages. This subsection will verify the effectiveness of multi-domain representations in localization performance. Figure 14 presents the maximum localization deviations of the four approaches under different combinations of representations. We think that the sole representation is single-faceted when expressing CSI, and thus, this experiment did not involve the localization performance of the sole representation. Therefore, the comparison representations are the combinations of every two groups, which are $R_{1}$ + $R_{2}$, $R_{2}$ + $R_{3}$, and $R_{1}$ + $R_{3}$. On the whole, compared to the other combinations, the fusion of the three representations achieves the best localization performance regarding the maximum deviation. It can be concluded that our design with three multi-domain representations plays an effective role in the proposed localization approach.

### 4.2. Experiment 2-Closed Laboratory

#### 4.2.1. Setup

**Figure 15:**
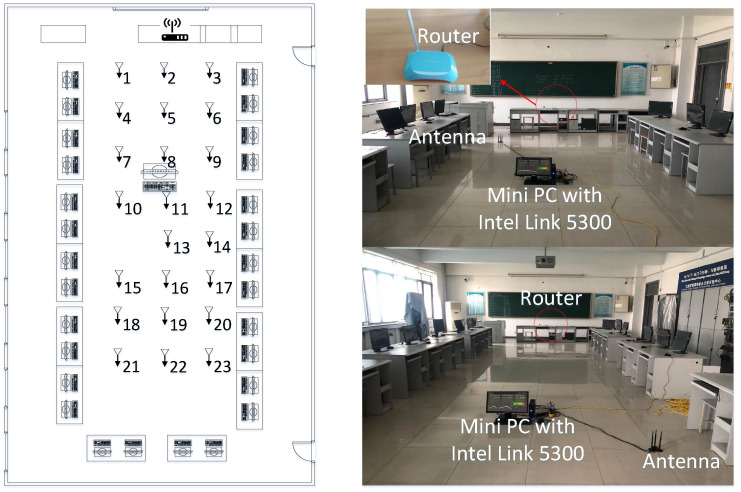
Experiment scene and planar graph of the laboratory.

Experiment 2 was conducted in an enclosed laboratory that was 7.5 m wide and 15 m long. Similarly, a TP-LINK router served as the transmitter and was placed on the table at the front of the lab. Meanwhile, a miniPC with antennas served as the receiver and was laid on the ground. Figure 15 presents the planar graph of the lab (left panel), where mobile antennas collected 23 fingerprints in the open space surrounded by computer desks, and the interval distance between two fingerprinting points was 1.2 m. On two consecutive days, we collected the CSI information of these 23 fingerprint points in periods $T_{1}$ and $T_{2}$, respectively. Here, period $T_{1}$ is earlier than period $T_{2}$. Nevertheless, we still conducted two-way experiments, where $T_{1}$-$T_{2}$ represents that the $T_{1}$ dataset is considered the source data domain and the $T_{2}$ dataset is considered the target data domain. Conversely, $T_{2}$-$T_{1}$ means the opposite.

#### 4.2.2. Localization Performance and Comparison

Table 2 lists the mean errors and the maximum errors of the four comparison methods, where LMDR-TCA was able to decrease the mean localization deviation by 49%, 65.9%, and 62% compared to LMDR-KNN, LMDR-SVM, and LMDR-GFK, respectively, under the $T_{1}$-$T_{2}$ condition, while the mean error of our method was almost one-third of those of the other methods under the $T_{2}$-$T_{1}$ condition.

**Figure 16:**
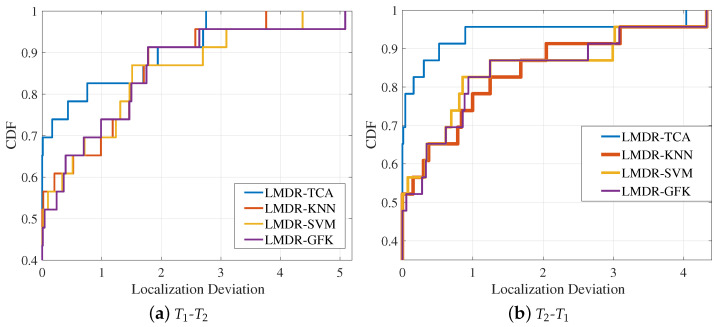
Cumulative distribution function of localization errors in the laboratory.

Figure 16 illustrates the CDF curves of the deviations of each fingerprinting point for the four approaches. LMDR-TCA was absolutely superior in terms of the localization accuracy under the error ranges of 1.5 and 3 m for the $T_{1}$-$T_{2}$ and $T_{2}$-$T_{1}$ conditions, respectively. When the error range was within 1 m, the localization accuracy (that is, the corresponding CDF value) of LMDR-TCA was approximately 13% better than those of the LMDR-KNN and LMDR-SVM and approximately 8.6% better than that of LMDR-GFK.

**Figure 17:**
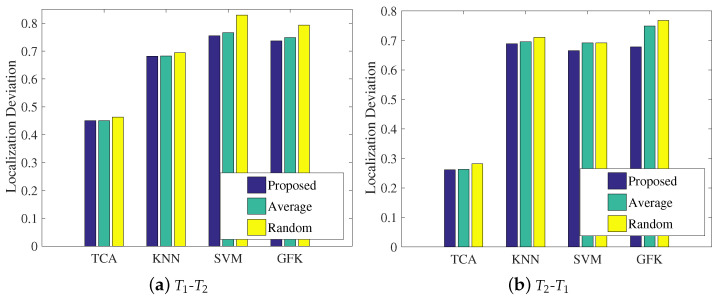
Impact of the weighted coefficient on localization performance in the laboratory.

As a supplement, we conducted some more experiments to thoroughly assess the impact of the weight coefficient on the localization performance in the closed scene. As seen in Figure 17, although the ranking order of the other compared methods is different from that in the open scenario, LMDR-TCA was always the best performer, and the proposed method of calculating the weight coefficients was effective at fusing the three classification results of the multi-domain representations in the final process of estimating the coordinates of the target point.

### 4.3. Experiment 3-Corridor

In this section, we present the localization results from experiments conducted on a smartphone platform in a corridor over several days.

#### 4.3.1. Setup

**Figure 18:**
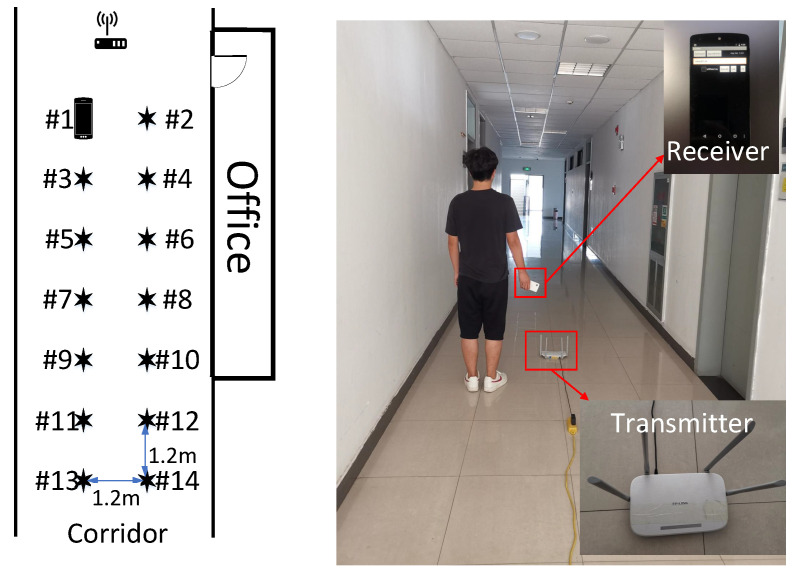
Experiment scene and planar graph of the corridor.

The experiment was implemented in a narrow corridor, as shown in Figure 18. This experiment’s hardware consisted of an off-the-shelf Wi-Fi router(TL-WDR5620, TP-Link) and a smartphone (Nexus 5, Google). We used Nexmon [33], a C-based firmware patching framework, to extract the CSI readings of Wi-Fi frames on the smartphone. To further evaluate the effectiveness and validity of the LMDR-TCA, we collected the CSI data from four conductive days, which were marked as $T_{1}$, $T_{2}$, $T_{3}$, and $T_{4}$. In each time period, we collected 14 fingerprint points, and each point lasted for 1 min (500 samples).

#### 4.3.2. Localization Performance over Time

**Figure 19:**
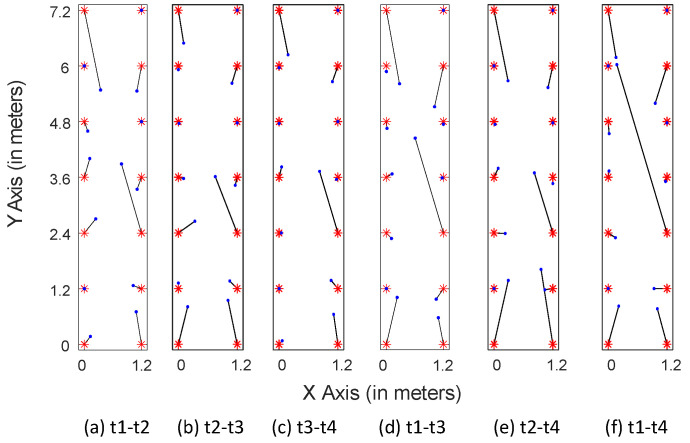
Localization results across different days.

**Figure 20:**
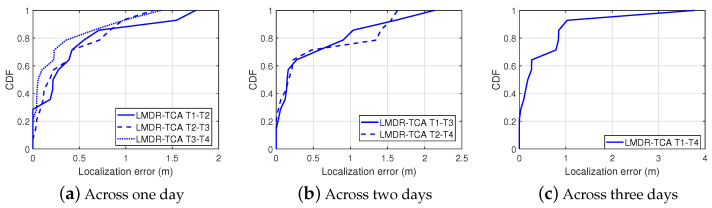
Cumulative distribution function of localization errors in the corridor.

Figure 19 shows the localization results of LMDR-TCA considering different time periods as the target and source domains. For instance, $T_{1}$-$T_{4}$ means that the data sampled at day 1 are used as the training dataset, whereas the data sampled at day 4 are used as the testing dataset. The red asterisks represent the ground truth points, the blue dots show the estimated results, and the black segments point toward the corresponding estimated positions from the real ones. To better show the localization results across different days, we divide the subfigures into three groups: localization results for spanning one day are shown in Figure 19a–c, two days in Figure 19d,e, and three days in Figure 19f. Obviously, localization across one day performs better than that across two days or three days, as the black segments in the first three subfigures are shorter than those in the last three subfigures. To be more specific, Table 3 lists the localization errors under six conditions and Figure 20 illustrates the cumulative distribution function of localization errors in three groups. It can be seen from the results that LMDR-TCA can achieve an average localization error of 0.3749 m when the testing data are one day after the training data, and an average error of 0.5153 m for across two-day localization; these results are comparable to those of experiment 2. Even when the testing data are three days apart from the training data, an average error of 0.5888 m can also be achieved for spanning three-day localization. Above all, it was indicated that LMDR-TCA performs well in localization across several days.

### 4.4. Analysis and Prospects

In this section, we would like to generalize the experimental results and discuss a few future issues.

(i) This paper proposes a novel indoor localization method for overcoming the problem of accuracy degradation because of time-varying Wi-Fi CSI readings. Experimental results verify that the proposed multi-domain representation mechanism plays a great role in improving localization accuracy, as with the fusion of the three alignment results after TCA, the max localization error decreases by up to 0.74 m compared to that obtained without using a combination of three representations.
(ii) We chose TCA to shorten the distribution differences of two time-varying CSI readings, achieving approximately 48%, 78%, and 22% increases in classification performance, on average, compared with other traditional methods (KNN and SVM) and another transfer learning method (GFK).
(iii) We also evaluated the proposed novel fusion mechanism for position estimation using a combination of the Bayesian model averaging and weighted centroid algorithms. The correlation coefficients of the three representation models served as the weight, which outperformed the methods using random weights and average weights.
(iv) In addition to a traditional computer platform based on an Intel Wi-Fi Link 5300 wireless network interface card, we also realized the experiments on a smartphone platform. Our method was proven effective even in an experiment that spanned three days.
(v) Regarding the first and second experiments, we found an interesting phenomenon in which the experimental results of $T_{2}$-$T_{1}$ were often better than those of $T_{1}$-$T_{2}$, whether in terms of the classification accuracy or positioning accuracy. The potential reason is the time difference in data collection. CSI values are influenced by many factors, such as multi-path effects, object obstacles, and even moving of targets upstairs or downstairs. These two groups of data may contain some noise affected by invisible factors, and we cannot tell which one is “cleaner”. We cannot ensure the relative stillness of an environment over time, and that is the reason that we explored a novel method for resolving this challenge in this paper. This interesting phenomenon may also inspire us to think about and research the impact of training data on the model performance and about how to judge the quality of training data.
(vi) Another interesting experimental result was also found: In experiment 1, positions #13 and #5 were confused in all three representations, but in different ways. One reasonable explanation is that points #13 and #5 were on the same line in the geography, and the attenuation paths and multiple paths might have been similar. Inspired by this, we could explore the impact of the similarity of different areas/positions on fingerprinting localization in the work.

## 5. Conclusions

This paper proposes a pioneering method for indoor fingerprinting localization by applying transfer learning to process dynamic CSI signals. First, this paper reveals the different distributions of CSI signals across time and uses CSI shape waves to calculate the similarity. We further combine the multi-domain representations of fine-grained subcarriers’ information and a transfer learning algorithm, which was named transfer component analysis, to design a robust and effective localization model, which was named LMDR-TCA, to overcome the problem of fingerprint variations over time. Two experiments were conducted in an open hall and a closed laboratory, where we collected two sets of CSI fingerprints over two days using a COTS Wi-Fi router. One group was defined as the labeled source data, and the other location was considered to be the target set that needed to be positioned. We collected CSI data from a computer equipped with an Intel WiFi Link 5300 wireless network interface card and conducted two experiments in an open hall and a laboratory. The proposed method’s effectiveness and robustness were verified by comparing the traditional CSI fingerprinting location method with different algorithm parameters. We also evaluated our method in terms of efficiency and validity of localization over several days on a smartphone platform. The experimental results show that the proposed system can achieve performance on a smartphone platform that is comparable with the performance on a computer platform and is characterized by good robustness.

## Figures and Tables

**Table 1:** Localization errors of the different methods.

	$T_{1}$-$T_{2}$				$T_{2}$-$T_{1}$			
LMDR-	TCA	KNN	SVM	GFK	TCA	KNN	SVM	GFK
Mean deviation	0.2227	0.3301	0.398	0.2737	0.0678	0.4335	0.3596	0.2362
Max deviation	1.6558	1.9187	1.911	1.9733	1.0525	2.5903	2.4034	1.6081

**Table 2:** Mean and maximum localization deviations among the different methods.

	$T_{1}$-$T_{2}$				$T_{2}$-$T_{1}$			
LMDR-	TCA	KNN	SVM	GFK	TCA	KNN	SVM	GFK
Mean deviation	0.4547	0.6814	0.7547	0.7367	0.2611	0.6887	0.6653	0.6783
Max deviation	2.7514	3.761	4.3716	5.0876	4.0353	4.3267	4.3267	4.3267

**Table 3:** Localization errors across different days.

Interval Days	One Day			Two Days		Three Days
Source-Target	$T_{1}$-$T_{2}$	$T_{2}$-$T_{3}$	$T_{3}$-$T_{4}$	$T_{1}$-$T_{3}$	$T_{2}$-$T_{4}$	$T_{1}$-$T_{4}$
Mean error (m)	0.4466	0.3855	0.2926	0.5132	0.5174	0.5888
Max error (m)	1.7514	1.2945	1.3788	2.1294	1.6323	3.7698

## Data Availability

The dataset is available on request from the authors.
